# Class effect for SGLT-2 inhibitors: a tale of 9 drugs

**DOI:** 10.1186/s12933-019-0899-9

**Published:** 2019-07-23

**Authors:** Dario Giugliano, Katherine Esposito

**Affiliations:** 1 Division of Endocrinology and Metabolic Diseases, Department of Advanced Medical and Surgical Sciences, Università della Campania Luigi Vanvitelli, University Hospital Luigi Vanvitelli, Piazza L. Miraglia 2, 80138 Naples, Italy; 2 Diabetes Unit, Department of Advanced Medical and Surgical Sciences, Università della Campania Luigi Vanvitelli, Naples, Italy

**Keywords:** SGLT-2 inhibitors, Class effect, MACE, Heart failure, Diabetic kidney disease

## Abstract

The definition of class effect for SGLT-2 inhibitors may be based on three concepts: a similar chemical structure, a similar mechanism of action and similar pharmacological effects. We have also assumed that a class effect does exist when an effect on a particular outcome is present and is significant for each drug within the class of SGLT-2 inhibitors. For major cardiovascular events (MACE), there is no class effect for SGLT-2 inhibitors, as the 7% reduction of MACE risk observed with dapagliflozin in the DECLARE trial was not significant; on the other hand, a class effect is evident for both heart failure and diabetic kidney disease, as in all four trials so far completed (EMPAREG-OUTCOME, CANVAS, DECLARE, CREDENCE) the risk of hospitalization for heart failure and progression of diabetic kidney disease was significantly reduced by all SGLT-2 inhibitors.

The role of the kidneys in glucose homeostasis was first described more than a century ago. Under normal conditions, the tubular glucose reabsorption is mediated by a sodium-dependent transport system consisting of a family of active glucose transporters: sodium–glucose transporter-2 (SGLT-2) mediate the reabsorption of about 90% of the filtered glucose, with the remainder occurring through SGLT-1. Within the context of normal renal function and in condition of normoglycemia, the vast majority of daily filtered glucose (approximately 200 g) is reabsorbed, because the overall SGLT-2-mediated process of renal glucose reabsorption has high capacity [1].

SGLT-2 inhibitors are a new class of orally active drugs used in the management of type 2 diabetes (T2D) [2]. The glucoside phlorizin (a type of flavonoid) was isolated from the bark of apple trees in 1835 by French chemists, and later (1886) the German physician von Mering demonstrated that phlorizin might cause glycosuria. Further research on this topic identified the active glucose-transport system located on the luminal membrane of proximal tubular cells (brush border). In 1987, phlorizin demonstrated its efficacy to reduce glycemic level in diabetic rats and to restore insulin sensitivity; however, low oral availability, intestinal side effects and short half-life hampered phlorizin use as a therapeutic agent [3].

Dapagliflozin was the first SGLT-2 inhibitor approved for the treatment of T2D (in Europe in 2012 and in the United States in 2014), followed by empagliflozin, canagliflozin, and ertugliflozin. Nowadays, SGLT-2 inhibitors, also known as the “gliflozins”, include other drugs such as ipragliflozin, luseogliflozin, and tofogliflozin, all launched in Japan in 2014, sotagliflozin, which has been approved in Europe for certain patients with type 1 diabetes with a body mass index of 27 kg/m^2^ or more, who could not achieve adequate glycemic control despite optimal insulin therapy, and lastly remogliflozin launched in India in April 2019 (Fig. 1). Given the unique mechanism of action, SGLT-2 inhibitors have potential for use as an adjunct therapy to enhance glucose lowering when used in combination with other glucose-lowering therapies.

**Fig. 1:**
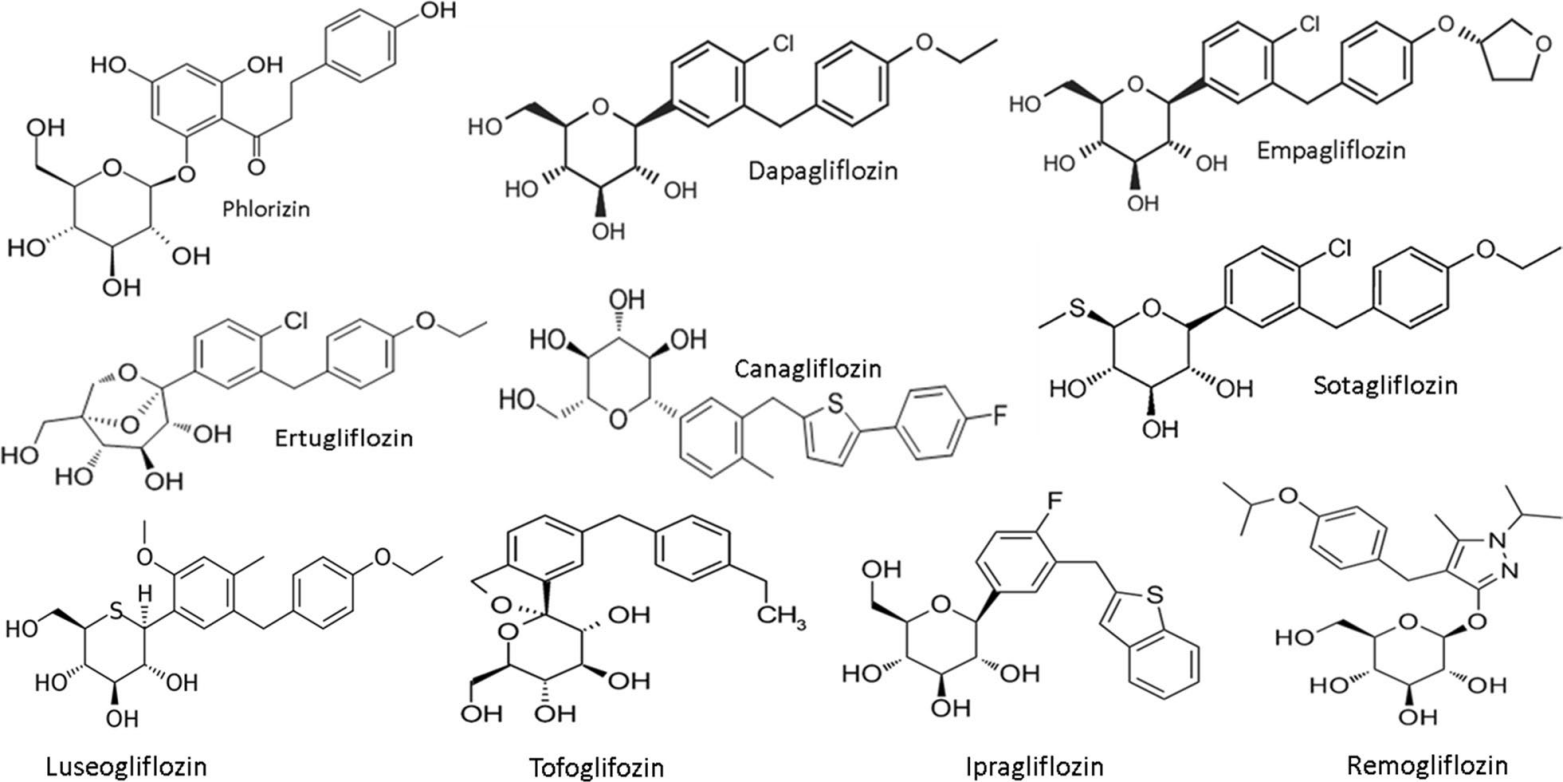
The class of SGLT-2 inhibitors with its founder phlorizin. Phlorizin-based analogs can be divided into *O*-glucoside analogs (the sugar group is bonded to another group via a glycoside bond, such the founder phlorizin and remogliflozin) or *C*-glucoside analogs (such as dapagliflozin and the many others); the latter have greater pharmacokinetic stability and selectivity for SGLT2. Inhibitor affinity is the result of a synergistic relationship between binding sites for sugar and the aglycone, with alterations in the sugar resulting in surprising differences in selectivity. All the nine SGLT-2 inhibitors showed in the figure are available in different parts of the world

On the basis of their ability to improve cardiovascular outcomes in high-risk individuals and slow the progression of diabetic kidney disease (DKD), SGLT-2 inhibitors should be considered reasonable second-line treatment options for individuals at risk of cardiovascular events or those with underlying nephropathy. Specifically, the American Diabetes Association [4] suggests to consider use of a SGLT-2 inhibitor in T2D patients with an estimated glomerular filtration rate ≥ 30 mL/min/1.73 m^2^ and particularly in those with > 300 mg/g albuminuria to reduce risk of diabetic kidney disease progression, cardiovascular events, or both. Adverse effects associated with SGLT-2 inhibitors include urinary frequency and dehydration. Other potential side effects include genitourinary tract infections and euglycemic diabetic ketoacidosis, while canagliflozin has been linked to lower-extremity amputations and bone fractures. In nationwide 2013–2016 registries from Denmark and Sweden, the use of SGLT-2 inhibitors, as compared with GLP-1 receptor agonists, was associated with an increased risk of lower limb amputation and diabetic ketoacidosis [5]. Moreover, the FDA warned about cases of necrotizing fasciitis of the perineum (i.e. Fournier’s gangrene which is a rare but potentially life-threatening affection) in patients treated with SGLT-2 inhibitors [6]. Although these adverse events [7] should not mask the overall cardiorenal benefits of SGLT-2 inhibitors [8], individuals at risk of these complications should be monitored closely and treatment should be reconsidered or discontinued if they occur.

## Class effect: which effect for which drug?

There are at least nine different SGLT-2 inhibitors available to treat diabetic hyperglycemia in different parts of the world. However, cardiovascular outcomes trials (CVOTs), a prerequisite for FDA approval of any new antidiabetic drug in order to exclude inacceptable cardiovascular burden for T2D patients [9], have been completed so far for some of them only, namely empagliflozin [10], dapagliflozin [11] and canagliflozin [12, 13]. Even limiting the focus upon these few trials (EMPAREG-OUTCOME, CANVAS, DECLARE, and for some instance CREDENCE), extrapolation of knowledge from one class member to another has become increasingly common [14], although the definition of the term “class effect” is not an easy one. Most pharmacological texts are silent on the matter, and there is no established regulatory definition of the term. It is possible that the term “class effect” represents a term of convenience, an heuristic device, rather than a necessary description of reality.

**Fig. 2:**
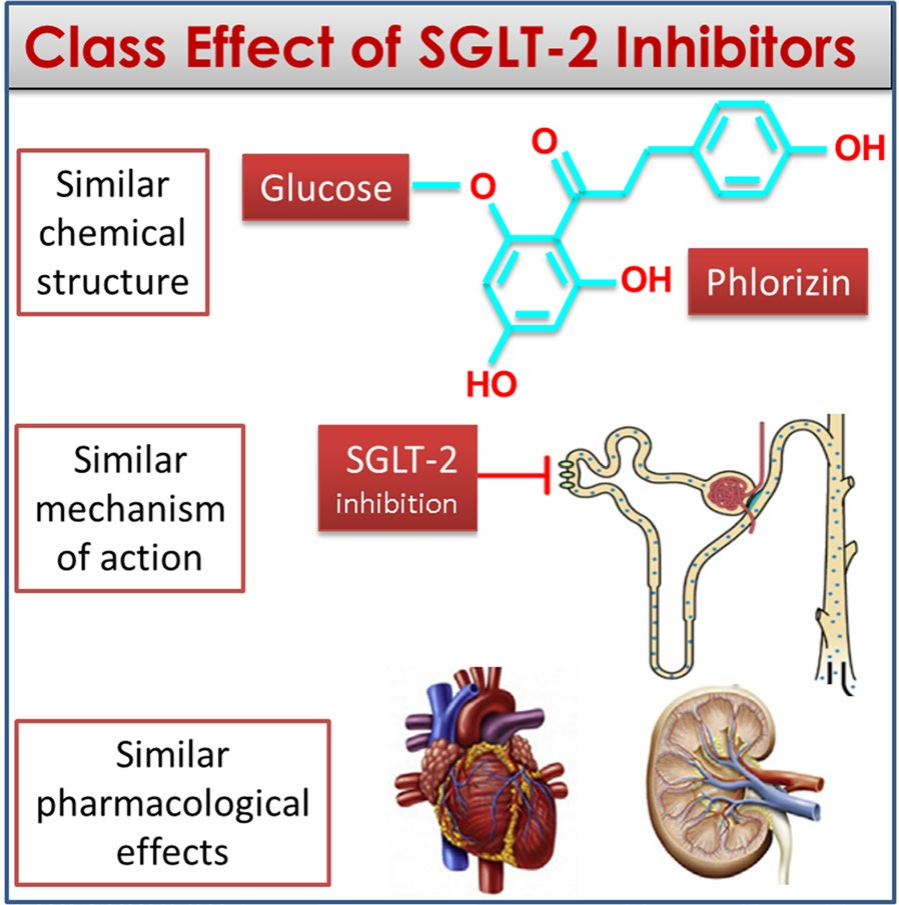
The three concepts that must be fulfilled for the definition of a class effect. Similar chemical structure, similar mechanism of action, similar pharmacological effects at the level of target organs; to all that, it must be added the statistical significance of the effect in each trial

The definition of drug class effect may be based on three concepts: a similar chemical structure, a similar mechanism of action and similar pharmacological effects [15]. A class effect is, therefore, an effect produced by all components of a chemically related group of drugs and not only by a single drug from that class [16]. Figure 2 shows the three concepts applied to SGLT-2 inhibitors: (a) they have a similar chemical structure derived by the founder phlorizin; (b) they have a similar mechanism of action based on the inhibition of the sodium-glucose transporter 2 predominantly expressed in the brush border membrane of the epithelial cells of the renal proximal tubule; and (c) they have similar pharmacological effects, at the level of the kidney (increase glycosuria and natriuresis, decrease glomerular pressure and albuminuria) and heart (decrease preload and afterload, increase ejection fraction). In order to make these concepts more stringent, we have assumed that a class effect does exist when an effect on a particular outcome is present and is significant for each drug within the class of SGLT-2 inhibitors [16].

**Fig. 3:**
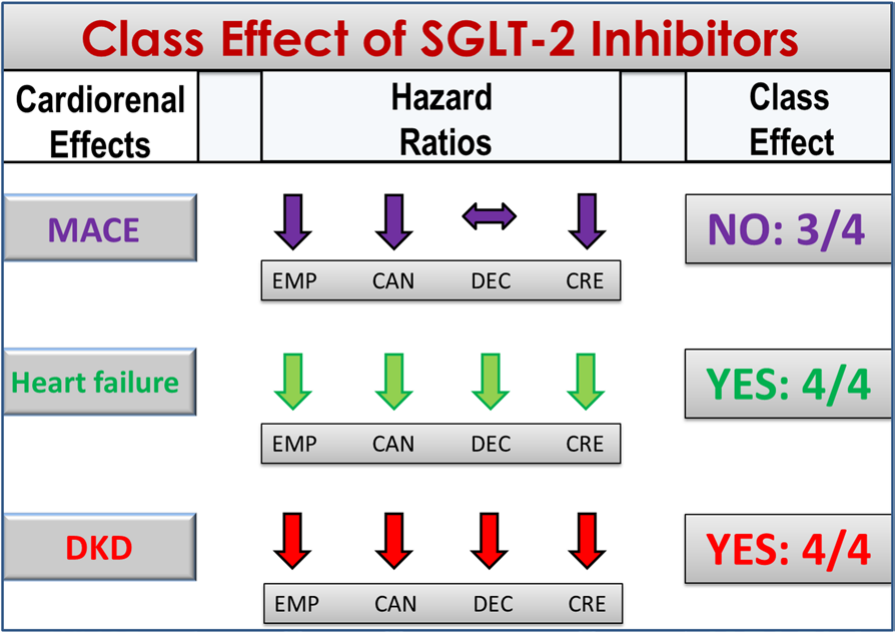
Class effects for the cardiorenal outcomes of SGLT-2 inhibitors. A class effect is not evident for MACE (only three trials demonstrated a significant reduction of the MACE risk), but is evident for heart failure (reduced risk of hospitalization for heart failure) and DKD progression (reduced decline of eGFR, renal death, or requirement for renal replacement therapy). MACE, major cardiovascular events; DKD, diabetic kidney disease; EMP, EMPAREG-OUTCOME; CAN, CANVAS; DEC, DECLARE; CRE, CREDENCE

Figure 3 shows the class effects of the three SGLT-2 inhibitors (empagliflozin, canagliflozin, dapagliflozin) according to the more stringent definition, as detailed above. The outcomes considered include MACE (major cardiovascular events), which was the primary outcome in EMPAREG-OUTCOME [10], CANVAS [12] and DECLARE [11], hospitalization for heart failure, which was a secondary outcome in all four trials, and progression of DKD, which was a secondary outcome in EMPAREG-OUTCOME [10], CANVAS [12] and DECLARE [11], and a primary outcome in CREDENCE [13]. The fraction indicates the number of trials that revealed a significant effect. For MACE, there is no class effect, as the 7% reduction of MACE risk observed in DECLARE [11] was not significant (HR = 0.93, 95% confidence interval, 0.84–1.03, P = 0.17); on the other hand, a class effect is evident for both hospitalization for heart failure and progression of DKD, as in all four trials the risk of HF and DKD progression was significantly reduced by all SGLT-2 inhibitors. Besides CVOTs, SGLT-2i use reduced hospitalization for heart failure by 36% compared with DPP-4i use in a real-world nationwide population-based cohort study [17]. Moreover, a beneficial effect of dapagliflozin on left ventricular diastolic functional parameters has been described in patients with heart failure after 6 months of treatment [18] and similar results have been found with canagliflozin after 3 months [19]; in addition, empagliflozin may improve arterial stiffness in patients with type 1 diabetes [20].

Although comparison between trials should always be done with caution, the four trials are consistent with each other, showing reliable cardiorenal benefit and comparable expected adverse effects. The proposed pharmacologic class effect is limited to the three SGLT-2 inhibitors reviewed in this paper (empagliflozin, canagliflozin, and dapagliflozin): it remains to be seen if it will extend to ertugliflozin, sotagliflozin (in Europe and USA for now) and other similar agents (luseogliflozin, ipragliflozin, tofogliflozin, remogliflozin) in other world regions.

## Conclusions

Up to 40% of individuals of the US adult diabetic population have some form of kidney disease [8]; moreover, overall cardiovascular disease affects approximately about 32% of all persons with T2D [21]. Until recently, the main treatment of diabetic kidney disease was with renin–angiotensin–aldosterone system inhibitors, and cardiovascular risk was mitigated primarily with statins and aspirin [22]. CVOTs of SGLT-2 inhibitors have consistently demonstrated cardiovascular risk reduction in the context of statin therapy, as well as slowing of kidney disease progression in the context of RAAS blockade therapy. Optimal prescribing of agents within this category requires a full understanding of their risks in addition to their benefits.

## Data Availability

Not applicable

## Abbreviations

SGLT-2: sodium–glucose transporter-2
T2D: type 2 diabetes
DKD: diabetic kidney disease
CVOTs: cardiovascular outcome trials
EMPAREG-OUTCOME: Empagliflozin Cardiovascular Outcome Event Trial in Type 2 Diabetes Mellitus Patients-Removing Excess Glucose randomized double-blind controlled
CANVAS: CANagliflozin CardioVascular Assessment Study
DECLARE: Dapagliflozin Effect on CardiovascuLAR Events randomized double-blind controlled trial
CREDENCE: Canagliflozin and Renal Events in Diabetes with Established Nephropathy Clinical Evaluation randomized double-blind controlled trial

## References

[1] Faillie J-L (2017). Pharmacological aspects of the safety of gliflozins. Pharmacol Res.

[2] Kramer CK, Zinman B (2019). Sodium–glucose cotransporter-2 (SGLT-2) inhibitors and the treatment of type 2 diabetes. Annu Rev Med..

[3] Ehrenkranz JR, Lewis NG, Kahn CR, Roth J (2005). Phlorizin: a review. Diabetes Metab Res Rev..

[4] American Diabetes Association (2019). Standards of Medical Care in Diabetes—2019. 11. Microvascular complications and foot care: American Diabetes Association. Diabetes Care..

[5] Ueda P, Svanström H, Melbye M (2018). Sodium glucose cotransporter 2 inhibitors and risk of serious adverse events: nationwide register based cohort study. BMJ.

[6] Tucker MA. FDA warns of serious genital infection with SGLT-2 inhibitors; 2018. https://www.medscape.com/viewarticle/901365. Accessed 29 Aug 2018.

[7] Scheen AJ (2019). An update on the safety of SGLT-2inhibitors. Expert Opin Drug Saf..

[8] Giugliano D, De Nicola L, Maiorino MI, Bellastella G, Esposito K (2019). Type 2 diabetes and the kidney: Insights from cardiovascular outcome trials. Diabetes Obes Metab.

[9] U.S. Food and Drug Administration. Guidance for industry: diabetes mellitus—evaluating cardiovascular risk in new antidiabetic therapies to treat type 2 diabetes; 2008. https://www.fda.gov/downloads/Drugs/GuidanceComplianceRegulatoryInformation/Guidances/ucm071627.pdf. Accessed 25 June 2019.

[10] Zinman B, Wanner C, Lachin JM (2015). Empagliflozin, cardiovascular outcomes, and mortality in type 2 diabetes. N Engl J Med.

[11] Wiviott SD, Raz I, Bonaca MP (2019). Dapagliflozin and cardiovascular outcomes in type 2 diabetes. N Engl J Med.

[12] Neal B, Perkovic V, Mahaffey KW (2017). Canagliflozin and cardiovascular and renal events in type 2 diabetes. N Engl J Med.

[13] Perkovic V, Jardine MJ, Neal B (2019). Canagliflozin and renal outcomes in type 2 diabetes and nephropathy. N Engl J Med.

[14] Katzung BG, Katzung BG (2014). Introduction. Basic and clinical pharmacology.

[15] Furberg CD (2000). Class effects and evidence based medicine. Clin Cardiol..

[16] Giugliano D, Meier JJ, Esposito K (2019). Heart failure and type 2 diabetes: from cardiovascular outcome trials, with hope. Diabetes Obes Metab.

[17] Kim YG, Han SJ, Kim DJ, Lee KW, Kim HJ (2018). Association between sodium glucose co-transporter 2 inhibitors and a reduced risk of heart failure in patients with type 2 diabetes mellitus: a real-world nationwide population-based cohort study. Cardiovasc Diabetol..

[18] Soga F, Tanaka H, Tatsumi K (2018). Impact of dapagliflozin on left ventricular diastolic function of patients with type 2 diabetic mellitus with chronic heart failure. Cardiovasc Diabetol..

[19] Matsutani D, Sakamoto M, Kayama Y, Takeda N, Horiuchi R, Utsunomiya K (2018). Effect of canagliflozin on left ventricular diastolic function in patients with type 2 diabetes. Cardiovasc Diabetol..

[20] Lunder M, Janić M, Japelj M, Juretič A, Janež A, Šabovič M (2018). Empagliflozin on top of metformin treatment improves arterial function in patients with type 1 diabetes mellitus. Cardiovasc Diabetol..

[21] Einarson TR, Acs A, Ludwig C, Panton CUH (2018). Prevalence of cardiovascular disease in type 2 diabetes: a systematic literature review of scientific evidence from across the world in 2007–2017. Cardiovasc Diabetol..

[22] Olesen KKW, Madsen M, Egholm G (2017). Patients with diabetes without significant angiographic coronary artery disease have the same risk of myocardial infarction as patients without diabetes in a real-world population receiving appropriate prophylactic treatment. Diabetes Care.

